# An extrahepatic hydatid cyst with the fat-fluid level

**DOI:** 10.1259/bjrcr.20210059

**Published:** 2021-11-26

**Authors:** Ferhat Yildirim, Aynur Turan, Selda Guven, Arda Ceylan

**Affiliations:** 1 University of Health Sciences, Diskapi Yildirim Beyazit Training and Research Hospital, Clinic of Radiology, Ankara, Turkey

## Abstract

A hydatic cyst is a zoonosis caused by the larva of a tapeworm *Echinococcus granulosus*. The liver is the most commonly affected organ. Soft tissue localization has been reported in 2.3% of cases. Herein, we present a patient with a fat-containing hydatid cyst located in the left thigh. There are only a few reports in the literature on the presence of the fat-fluid level within a hydatid cyst. Previous studies have suggested that fat-containing hydatid cysts occur due to their cysto-biliary communication in the liver. In our case, we describe a fat-containing hydatid cyst in the extrahepatic location and discuss the pathophysiologic mechanism of fat inside it.

## Introduction

Hydatid disease is a global endemic parasitic disease produced by the larval stage of the *Echinococcus* tapeworm*s*. *Echinococcus granulosus* is the most frequently encountered type of hydatid disease in humans. Echinococcosis cysts are found in the liver in approximately 75% of cases, but they can be located anywhere in the body.^1^ Soft tissue localization with the fat-fluid level is rarely described in the literature. This report presents a patient with a fat-containing hydatid lesion in the extrahepatic location and discusses the pathophysiologic mechanism.

## Case report

**Figure 1.**
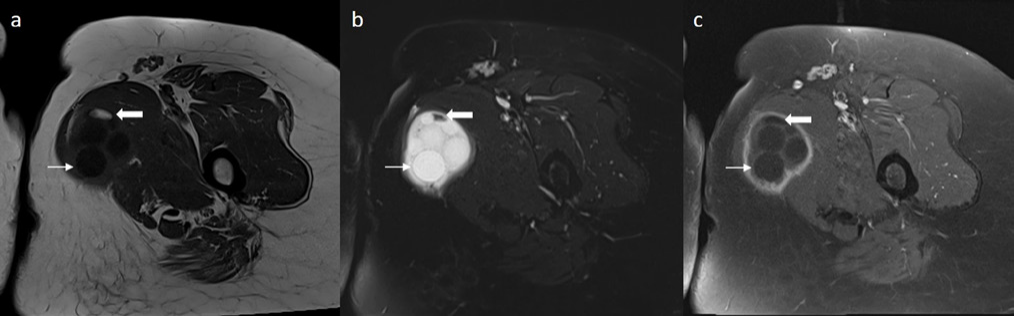
Axial *T*_1_-weighted image (**a**) demonstrates a hypointense lesion with T1 hyperintense area (thick arrow) in the medial part of the left thigh. *T*_2_-weighted fat-saturated (**b**) and post-contrast fat-saturated *T*_1_-weighted (**c**) confirm macroscopic fat and fat-fluid level in the lesion, by showing signal loss in aftermentioned areas (thick arrow). Multiple daughter vesicles are also demonstrated (arrow).

**Figure 2.**
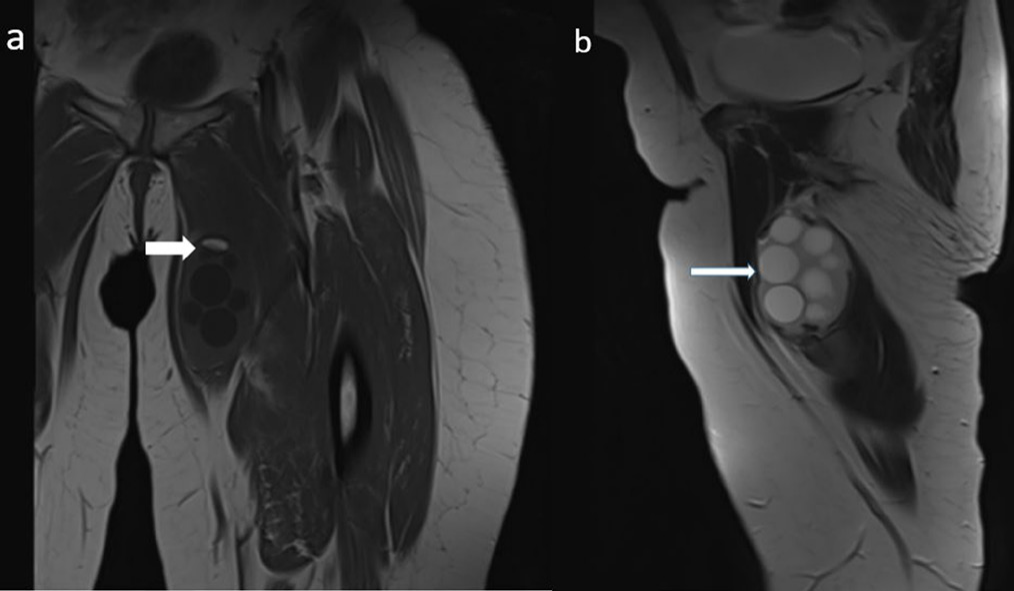
Coronal *T*_1_-weighted image (**a**) demonstrates fat component within the lesion (thick arrow). Sagittal *T*_2_-weighted image (**b**) shows multiple cyst, compatible with daughter vesicles (arrow).

A 54-year-old female presented with a palpable mass and swelling in the upper leg. She stated that she had palpable swelling at this level for a long time, but it became apparent for about a month. A well-circumscribed heterogeneous hypoechoic cystic lesion on the medial side-of the left thigh was observed on ultrasonographic examination. Afterwards, magnetic resonance imaging (MRI) revealed a cystic lesion with daughter vesicles and the fat-fluid level in the medial part of the left thigh (Figures 1 and 2). The indirect hemagglutination test for cyst hydatid was positive. Serological examination and radiological findings were evaluated together, and the patient was diagnosed with a hydatid cyst. The lesion was excised, and medical treatment (Albendazole) was applied. No post-operative residual lesion was detected on ultrasonography, and the patient was discharged.

## Discussion

Hydatid disease is a global zoonosis produced by the larval stage of the *Echinococcus* tapeworm. *Echinococcus granulosus* and *Echinococcus multilocularis* are the two main types of hydatid disease. Hydatid disease is widespread in many areas worldwide, but it is endemic in the Middle East, Africa, South America, and Australia. Humans are infected by the ingestion of food or water contaminated by dog stool containing the parasite’s eggs. The patient’s symptoms depend on the location and size of the cyst and usuallydue to the cyst’s compressive effect.^1^

The liver is the most commonly affected organ in hydatid disease. A soft tissue hydatid cyst is seen in 2.3% of the patients in endemic regions. This low incidence in soft tissue is because of the cyst’s difficulty growing inside the muscle due to lactic acid and muscle contraction.^1^

A hydatid cyst can present with various findings and signs. The radiological appearance of hydatid disease ranges from a purely cystic to a completely solid lesion, depending on the stage of cyst growth, maturation, and degeneration. In addition, it can be partially or entirely calcified. It may also contain fat globules and fat-fluid levels, which are rarely described in the literature. There are two theories to explain the presence of fat in hydatic cysts. First, Montero et al suggested that the fat within the cyst was generally caused by the rupture or erosion of the cyst into the biliary system^2^; therefore, the presence of lipid material in the cyst was an indirect finding of biliary communication.

Nonetheless, the biliary communication of the cyst changes the treatment options of these patients. In uncomplicated cysts, percutaneous ultrasonography-guided drainage techniques such as puncture-aspiration-injection-reaspiration and other treatment options include catheterization techniques involving the injection of scolicidal agents such as alcohol. However, surgery is the preferred treatment in complicated cyst cases of cysto-biliary communication and rupture.^3^ Hence, it is essential to determine whether there is a rupture of the biliary system. The second theory concerns cyst degeneration and maturation. Beric and Blomley stated that the fat-fluid level within the cyst was related to the degeneration of hydatid membranes since histopathological and biochemical evidence suggest that lipids play an essential role in metabolism of hydatid disease.^4^ This is supported by the fat-fluid level within the hydatid cyst outside the liver in our case. It may indicate that the fat found within the hepatic hydatid cyst can be related to its maturation and degeneration. This should be taken into account when considering the patient’s treatment options.

In the literature, fat-containing hydatid cysts are generally seen in the liver, but soft tissue localization is not found. Therefore, we consider that our case will contribute to the literature.

The differential diagnosis of fat-containing cystic lesions includes mature cystic teratoma, dermoid cyst, epidermoid cyst, and fat necrosis.^5,6^ However, the treatment preferences of these lesions are not the same, and to prevent possible complications, it is critical to consider the possibility of a hydatid cyst in the differential diagnosis of fat-containing lesions.

In conclusion, the presence of fat within a hydatid cyst is a rare finding, most probably related to the maturation and degeneration of hydatid membranes. This information can change the treatment approach to fat-containing hydatid cysts of the liver, and it is also essential for the differential diagnosis of fat-containing cystic lesions located in other parts of the body to prevent possible complications.

## Learning Points

1. Hydatic cysts can be encountered in any organ in endemic regions.
2. Hydatic cysts can contain fat even in the extrahepatic locations without biliary connection.
